# Distinct transcriptomic signatures discriminate hyperinflammation from immune paralysis in sepsis: a single-cell RNA sequencing study

**DOI:** 10.1186/s13054-026-06229-w

**Published:** 2026-08-01

**Authors:** Inge Grondman, Valerie A. C. M. Koeken, Tristan Couwenbergh, Athanasios Karageorgos, Wenchao Li, Nikolaos Antonakos, Bowen Zhang, Georgia Damoraki, Wouter A. van der Heijden, Cheng-Jian Xu, Evangelos J. Giamarellos-Bourboulis, Yang Li, Mihai G. Netea

**Affiliations:** 1 Department of Internal Medicine and Radboud Center for Infectious Diseases, Radboud University Medical Center, Geert Grooteplein 8, Nijmegen, 6525GA The Netherlands; 2 Department of Computational Biology for Individualised Medicine, Centre for Individualised Infection Medicine (CiiM), A Joint Venture Between the Helmholtz Centre for Infection Research (HZI) and the Hannover Medical School (MHH), Hannover, Germany; 3 TWINCORE, Centre for Experimental and Clinical Infection Research, A Joint Venture Between the Helmholtz Centre for Infection Research (HZI) and Hannover Medical School (MHH), Hannover, Germany; 4 Research Centre Innovations in Care, Rotterdam University of Applied Sciences, Rotterdam, The Netherlands; 5 4th Department of Internal Medicine, National and Kapodistrian University of Athens Medical School, Athens, Greece; 6 Department of Intensive Care and Radboud Center for Infectious Diseases, Radboud University Medical Center, Nijmegen, The Netherlands; 7 Hellenic Institute for the Study of Sepsis, Athens, Greece; 8 Department of Immunology and Metabolism, Life and Medical Sciences Institute (LIMES), University of Bonn, Bonn, Germany

**Keywords:** Sepsis, Hyperinflammation, Immune paralysis, Single-cell RNA sequencing

## Abstract

**Background:**

Sepsis is a highly heterogeneous syndrome characterized by variable immune dysregulation states, including hyperinflammation and immunosuppression. Previous immunotherapy attempts in sepsis have largely failed, likely due to a “one-size-fits-all” approach that ignores each patient’s immune status. The recent ImmunoSep randomized clinical trial demonstrated that precision immunotherapy guided by the presence of either macrophage activation–like syndrome (MALS) or immune paralysis can improve early organ dysfunction in sepsis patients. However, the molecular mechanisms underlying these immune endotypes remain unclear.

**Objectives:**

To identify the immunological signatures that distinguish MALS and immune paralysis.

**Methods:**

We used single-cell RNA sequencing to profile circulating leukocytes of 6 healthy controls and 16 sepsis patients classified as MALS, immune paralysis or unclassified (when criteria for neither of these two immune endotypes were applicable). Classification was based on surrogate biomarkers ferritin and HLA-DR expression on monocytes. Thereafter, the transcriptional programs of these groups were compared.

**Results:**

Pronounced differences were detected mainly in the transcriptional signature of monocytes from these patients, with a clear distinction between MALS and immune paralysis. Unsupervised clustering analysis revealed the existence of MALS-specific monocyte clusters, as well as one sepsis-specific monocyte cluster that was linked to greater comorbidity burden and may reflect increased clinical vulnerability in sepsis. These findings were validated in two independent cohorts, in which urosepsis was characterized by heterogeneous MALS and immune paralysis monocyte signatures. Moreover, MALS-specific monocyte clusters showed overlapping transcriptional signatures with severe COVID-19.

**Conclusions:**

Our findings shed light on the heterogeneous immune landscape underlying sepsis and provide opportunities for patient stratification for future therapeutic development.

**Supplementary Information:**

The online version contains supplementary material available at https://doi.org/10.1186/s13054-026-06229-w.

## Background

Sepsis is a life-threatening disease in which a dysregulated host response to infection is a crucial component of the pathogenesis [1–3].

Different immunological and clinical phenotypes can be recognized in sepsis. Some patients develop an overwhelming hyperinflammatory response, particularly during early stages of disease. This state has been termed macrophage activation-like syndrome (MALS) [4, 5]. MALS is associated with high mortality, and elevated circulating ferritin concentrations (> 4,420 ng/mL) have been proposed as an important biomarker for this immune endotype [4]. In addition, sepsis patients may also develop a profound state of immunosuppression known as immune paralysis, characterized by reduced expression of monocytic HLA-DR and clinically recognized by increased vulnerability to opportunistic infections [6–8].

Sepsis heterogeneity is considered to be a major barrier to effective immune-based treatment, which may explain the failure of previous immunomodulatory trials [9–11]. Therefore, identifying patient endotypes with distinct immune dysregulation signatures offers a promising approach for personalized treatment strategies. Multiple studies have performed subtyping in sepsis, either based on transcriptomics [12, 13] or biomarkers [14–16], linking immune responses to patient outcomes. While these approaches have improved the stratification of sepsis, they did not lead to new therapeutic targets or positive outcomes in clinical trials.

A recent study has shown that high ferritin circulating concentrations and decreased monocytic HLA-DR expression can be used to characterize patients with distinct features of MALS or immune paralysis [17]. Based on these novel insights, a strategy of precision immunotherapy based on patient stratification into the MALS and immune paralysis endotypes was used in the multicenter double-blind randomized ImmunoSep phase 2 clinical trial. Patients with MALS were treated with anakinra and patients with immune paralysis received IFN-γ. This trial showed a significant improvement in early organ dysfunction in sepsis compared to placebo [18].

Despite these advances, the molecular mechanisms underlying MALS and immune paralysis remain poorly understood. Although single-cell transcriptomic studies have proven useful in mapping immune cell states and functional programs in sepsis [19–21], no previous studies have specifically characterized the cellular and transcriptional mechanisms of MALS and immune paralysis at single-cell level. This represents an important knowledge gap, because these immune endotypes are increasingly used to guide directed immunotherapy. Therefore, we profiled the transcriptome of circulating immune cells at the single-cell level to identify immunological signatures that distinguish MALS and immune paralysis. This approach has the potential to further facilitate personalized care and pre-enrichment strategies in sepsis patients and identify new immunotherapeutic approaches.

## Methods

### Study design

This study includes samples of a subset of sepsis patients who participated in a double-blind, double-dummy randomized clinical trial that took place between November 2017 and December 2019 in Greece, called the PROVIDE study (ClinicalTrials.gov identifier NCT 03332225, EudraCT number 2017-002171-26). The PROVIDE study consisted of one screening/observational stage and one intervention (clinical trial) stage [17]. Samples for this study were collected during the screening stage.

### Patient population and inclusion

#### Subjects

The sepsis patients participating in this study were recruited to one of the 14 study sites in Greece, meeting inclusion and exclusion criteria as described before [17]. All patients (newly admitted or already hospitalized) included were diagnosed with sepsis or septic shock, according to the Sepsis-3 definition [2], due to community-acquired pneumonia, healthcare-associated pneumonia, ventilator-associated pneumonia, acute cholangitis, or primary bloodstream infection. These infections were defined according to internationally used criteria [22] and were applied to all patients, as previously described [17]. In parallel, control samples were collected from middle-aged healthy individuals without known comorbidities or chronic diseases, and no use of medication.

#### Immunological screening procedures

Patients diagnosed with sepsis due to lung, primary bacteremia, or acute cholangitis were subject to additional screening methods for their immune state. Blood samples were obtained through venipuncture; within the first 24 h after diagnosis (day 1) and this was repeated after 24 h (day 2). Samples were collected in EDTA-tubes and transferred via courier to the central laboratory. Ferritin was measured daily in serum by an enzyme immunosorbent assay (ORGENTEC Diagnostika GmbH, Mainz, Germany; lower detection limit 75 ng/mL). Using whole blood from the EDTA vial, white blood cells were incubated for 15 min in the dark with the following monoclonal antibodies: Quantibrite HLA-DR/anti-monocyte PerCP-Cy5 (Becton Dickinson, Cockeysville Md). After incubation, cells were analyzed by flow cytometer FC500 versus cells stained with anti-CD45 PC5 and anti-idiotypic IgG1. HLA-DR molecules on CD14/CD45 monocytes were expressed as a number of molecules per cell.

#### Subgroup definitions

According to the immune classification that was proposed in this study [17], MALS was identified in patients with ferritin concentration > 4,420 ng/mL, which was measured on the day on which samples could be obtained (day 1 or day 2), regardless of flow cytometry results. Immune paralysis was defined as HLA-DR/CD14/CD45 expression < 5,000 Mab/cell and ferritin concentration was ≤ 4,420 ng/mL. Patients that could not be classified as MALS or immune paralysis were considered having an intermediate immune status (ferritin ≤ 4,420 ng/mL and HLA-DR ≥ 5,000 antibodies/monocyte), so-called “unclassified”.

#### Patient characteristics

Baseline characteristics, clinical data (including source of infection, comorbidities and calculated CCI), and outcome were collected for each patient from medical files. The Acute Physiology and Chronic Health Evaluation (APACHE II) and the Sequential Organ Failure Assessment (SOFA) score were calculated on day 1.

### PBMC isolation and cryopreservation

Peripheral blood mononuclear cells (PBMCs) were isolated from 20 mL EDTA blood samples that were obtained in parallel with immunological screening procedures (day 1 or 2), using optimized density 1.077 g/mL (Lymphosep, Biowest) gradient centrifugation. After three washings in ice-cold PBS (phosphate buffered saline, pH: 7.2), PBMCs were resuspended in RPMI 1640 (Biochrom, Berlin, Germany) enriched with 2 mM of L-glutamine, 100 U/mL penicillin G, 0.1 mg/mL streptomycin. Cells were counted using a Neubauer plate with trypan blue exclusion of dead cells. Next, cells were cryopreserved with a density of 10 × 10^6^/mL per vial in freeze medium containing 90% FBS and 10% DMSO by using a freezing container (Coolcell) at -80 °C overnight. Thereafter, vials were transferred for long-term storage to liquid nitrogen, until further analysis.

### DNA genotyping

DNA was isolated from 0.5 mL blood with the ReliaPrep chemistry (Promega) automated on the Freedom Evo Robot (Tecan). Next, genotyping was performed using the GSA-MDv3 array (Infinium, Illumina) with a lab-specific clusterfile. This information on genetic variation was used for demultiplexing of the single-cell RNA-sequencing data.

### Single-cell RNA-sequencing and downstream analyses

#### Sample preparation and sequencing

Frozen PBMCs were thawed according to the instructions given by 10x Genomics in preparation for their Single Cell protocols (Fresh frozen human peripheral blood mononuclear cells for single cell RNA sequencing protocol- Document CG00039). Briefly, frozen PBMCs were recovered by rapidly thawing of cell suspensions at 37 °C and immediately sequential dilution (1:1) with pre-warmed RPMI media containing 40% fetal bovine serum (FBS). The dilution step was repeated every minute for a total of 5 times, followed by centrifugation at 300 g for 5 min. After centrifugation, cells were resuspended in PBS with 0.04% BSA, washed once, passed through a 40 μm mesh cell strainer, followed by determination of cell concentrations and viability using an automated Cell counter (TC20 automated Cell counter, Bio-rad). Next, samples were prepped for scRNA-seq according to the 10X Genomics Chromium™ Single Cell 3′ v2 RNA sequencing specification. Cells from 3 to 5 samples were pooled for one reaction at a final stock within the recommended range (700–1200 cells/µL) and loaded at a volume with a targeted cell recovery count of 1200 cells for each sample. As a high percentage of non-viable cells may impact the targeted cell recovery in these pools, in case the minimum viability (≥ 70%) was not achieved, non-viable cells were first removed from the individual samples with a removal of dead cell kit according to the instructions and recommendations of the manufacturer and 10x Genomics (MACS Dead Cell Removal Kit, Miltenyi; removal of dead cells for single cell RNA sequencing-document CG000093) before pooled and loaded. The generated cDNA was used for Illumina next-generation sequencing using a NextSeq500-v2 150 cycle kit with a sequencing depth of 25,000 reads/cell.

#### Reads alignment and demultiplexing

The data were aligned to the GRCh38 reference genome using CellRanger (10X Genomics) to generate count tables. Then, demultiplexing of the samples within each pool was performed using souporcell, which is a method to cluster cells using the genetic variants detected within the scRNA-seq reads [23]. Doublets detected by souporcell were excluded from further analysis.

#### Quality control, unsupervised clustering and cell annotation

After demultiplexing, samples were further analyzed using Seurat v3 [24] in R version 4.0.3. Cells were excluded if the number of unique genes expressed was < 200 or > 4000 and if the percentage of mitochondrial reads was > 25%. Log-normalization was applied before downstream analysis and the count data was scaled by regressing for the percentage of mitochondrial reads. Mitochondrial and ribosomal reads were also excluded from downstream analysis. Subsequently, principal component analysis was performed using the top 2000 variable genes as input. Then, the first 20 dimensions were used as input for Uniform Manifold Approximation and Projection (UMAP) to cluster cells in an unsupervised manner. Clusters were identified using a shared nearest neighbor (SNN) modularity approach with a resolution of 0.4. These clusters were then annotated to their respective cell types based on the expression of canonical markers.

#### Cell proportion, differential expression and pathway enrichment analysis

Cell proportion differences were calculated using Dirichlet-multinomial regression. Then, per cell type, differentially expressed genes were identified using a Wilcoxon rank-sum test, while no filtering was performed based on the fold difference. These differentially expressed genes were then used for KEGG enrichment analysis separately for up and downregulated genes using the R package clusterProfiler [25]. Throughout the study, p-value adjustment was performed using FDR, except for the differential expression analyses, where a Bonferroni correction was applied based on the recommendations from the Seurat R package [24]. A p-value < 0.05 was considered statistically significant and all tests performed were based on two-sided hypothesis testing.

#### Cell-cell interaction network

A ligand-to-target interaction network was generated based on genes differentially expressed in sepsis compared to healthy controls. The NicheNet algorithm was used with monocytes as the receiver cell type and all the other main cell types as sender cells [26]. The ligand-receptor interactions were visualized using the circlize package in R [27].

#### Identification of regulatory transcription factors

To identify the transcription factors regulating the expression profiles of monocytes from the three endotypes in sepsis, we followed the SCENIC workflow [28]. The RcisTarget and GENIE3 R packages were used to identify regulons and to reconstruct gene regulatory networks [29]. The hg38 (refseq_r80) motifs version 9 database was used to annotate to transcription factors. The genes that were differentially expressed in monocytes between the three endotypes (MALS, unclassified and immune paralysis) were used as target genes.

#### Signature genes of monocyte clusters and gene set enrichment analysis

Due to the heterogeneity in the monocyte population in this study, we selected only the monocytes and performed UMAP on the first 20 dimensions with a resolution of 0.2 to identify clusters within the monocyte population. The marker genes for each cluster were identified using Wilcoxon rank-sum tests, while being expressed in at least 25% of the cells of a cluster with a log fold difference threshold of 0.25. The top 10 marker genes for each monocyte cluster were subsequently visualized in a heatmap. A gene set enrichment analysis was performed using the R package AUCell [28] to test the enrichment of signatures of an in-vitro LPS tolerance model, bacterial sepsis [30] and severe COVID-19 [31] in the marker genes of the monocyte subsets identified in this sepsis cohort. The area under the curve (AUC) values were subsequently visualized in violin plots.

### *In-vitro* LPS tolerance model

#### Sample preparation and stimulation

Isolation of monocytes and induction of immune tolerance by LPS was performed as earlier described [32]. Briefly, PBMCs from three anonymous healthy donors were isolated by density centrifugation of Ficoll-Paque (GE healthcare, UK), followed by monocyte enrichment by Percoll gradient. These donors were distinct from the healthy controls included in the main single-cell RNA-sequencing cohort. Cells were resuspended in RPMI 1640 culture medium (Roswell Park Memorial Institute medium; Invitrogen, CA, USA) supplemented with 2 mM Glutamax (GIBCO), 1 mM pyruvate (GIBCO), and 50 µg/mL gentamicin. After one hour of adherence, the non-adherent cells were washed away with PBS, and the monocytes were incubated with culture medium only (negative control) or 10 ng/mL LPS for 24 h at 37 °C. Then, cells were washed with PBS and incubated for 5 days in culture medium supplemented with 10% human pooled serum, after which the monocytes were restimulated with 10 ng/mL LPS at day 6 for 4 h.

#### Cell sorting and single-cell RNA-sequencing

Monocytes were sorted from all samples based on forward and side scatter, and well-based scRNA-seq using SORT-seq platform was performed, which is based on the CEL-Seq2 protocol [33, 34]. Viable single cells were sorted into 192 or 384-well plates and transcriptome of each well containing a single cell was sequenced.

#### Reads alignment and quality control

First, paired-end reads were processed with the SORT-seq pipeline. Fastq reads were aligned to the human transcriptome (GRCh38) with the Burrows-Wheeler Alignment tool [35]. The output of this pipeline is a digital gene expression (DGE) matrix for each sample, which records the number of unique molecular identifiers (UMIs) for each gene that are associated with each cell barcode. Genes detected in less than three cells were removed from the DGE matrix. Cells were also filtered if the number of detected genes was less than 100 or more than 7000, to avoid empty wells or doublets, and if more than 25% of the gene counts were from mitochondrial genes, to remove dying cells.

#### Unsupervised clustering, cell annotation and differential expression analysis

Seurat v3.1 integration workflow with SCTransform normalization method was used to cluster cells into distinct cell subsets [36]. In order to keep the biological differences for downstream analyses, batch correction was only used in the cell clustering related steps. For the other analyses, we used standard LogNormalization methods. Then, differentially expressed genes were identified using FindMarkers/FindAllMarkers functions in Seurat v3.1 with the Wilcoxon rank-sum test. For each comparison, genes expressed in at least 10% of the cells per group and Bonferroni corrected P-values < 0.05 were regarded as significantly differentially expressed genes. The gene signature of LPS tolerance was defined as genes detected by differential expression analysis between LPS-restimulated monocytes of tolerized monocytes (primed with LPS) versus LPS-restimulated monocytes of RPMI-controls (primed by RPMI) in the in-vitro experiments.

### Statistics

The statistical analyses of the baseline and clinical characteristics were performed using Kruskal-Wallis tests for continuous variables, and Fisher-Freeman-Halton tests for nominal variables. Data normality was not formally tested, as continuous variables were assumed to follow a non-Gaussian distribution because of the small sample size and are therefore presented as median with interquartile range. A p-value < 0.05 was considered statistically significant and Bonferroni correction was applied to correct for multiple testing. The analysis of the single-cell RNA-sequencing is described in detail (see the two previous sections). All statistical analyses were performed using IBM statistics, SPSS version 25 or R/Bioconductor (R version 4.0.3; https://www.R-project.org/).

## Results

### Sample overview and cell proportion changes in sepsis immune endotypes

Baseline and clinical characteristics of included subjects are summarized in Table 1. Overall, no substantial differences in patient characteristics were observed between subgroups, except for a significantly higher Sequential Organ Failure Assessment (SOFA) score in the MALS subgroup, compared to patients with immune paralysis and those defined unclassified. The healthy individuals (*n* = 6; 4 females, 2 males) had a slightly lower age (median of 52 years [IQR 44–56] versus 66 years in the total sepsis population [IQR 59–85], p-value 0.013). Although not statistically significant, the sex distribution differed between healthy controls and sepsis patients, with a higher proportion of females among healthy controls and a higher proportion of males among sepsis patients.

**Table 1:** Baseline and clinical characteristics of healthy controls, sepsis patients and the immunological subgroups

	Healthy controls (*n* = 6)	Total sepsis (*n* = 16)	MALS (*n* = 5)	Immune paralysis (*n* = 5)	Unclassified (*n* = 6)	*P*-value*
Age, years	52 (44–56)	66 (59–85)	65 (62–80)	65 (45–91)	72 (45–81)	0.013^†^
Gender (n,%) Male Female	2 (33) 4 (67)	12 (75) 4 (25)	4 (80) 1 (20)	3 (60) 2 (40)	5 (83) 1 (17)	NS NS
Body Mass Index, kg/m^2^	27.2 (24.4–32.1)	27.1 (21.6–29.4)	29.4 (22.0-35.3)	21.5 (21.0-25.9)	27.2 (25.4–29.4)	NS
Comorbidities (n,%) Diabetes mellitus Heart failure Coronary heart disease Chronic renal disease Chronic obstructive pulmonary disease History of hematological/solid malignancies	NA NA NA NA NA NA	6 (38) 5 (31) 7 (44) 1 (6) 5 (31) 2 (13)	2 (40) 1 (20) 3 (60) 0 (0) 1 (20) 1 (20)	2 (40) 1 (20) 0 (0) 0 (0) 1 (20) 1 (20)	2 (33) 3 (50) 4 (67) 1 (17) 3 (50) 0 (0)	NS NS NS NS NS NS
Charlson comorbidity index (CCI)	NA	5 (3–8)	5 (4–6)	6 (1–7)	7 (3–8)	NS
Source of infection (n,%) Acute cholangitis Primary bacteremia Community acquired pneumonia Health care associated pneumonia Ventilator associated pneumonia	NA NA NA NA NA	1 (6) 2 (13) 6 (38) 4 (25) 3 (19)	1 (20) 1 (20) 0 (0) 3 (60) 0 (0)	0 (0) 1 (20) 3 (60) 0 (0) 1 (20)	0 (0) 0 (0) 3 (50) 1 (17) 2 (33)	NS NS NS NS NS
Septic shock (n,%)	NA	14 (88)	5 (100)	4 (80)	5 (83)	NS
SOFA score	NA	12 (8–15)	15 (15–17)	8 (6–14)	11 (8–12)	**0.009** ^‡^
Mortality 28 days (n,%)	NA	4 (25)	3 (60)	0 (0)	1 (17)	NS

Data are presented as median with interquartile range (IQR) or n (%)

*The difference between 2 groups (healthy controls vs sepsis) was statistically tested with the Mann-Whitney U test for continuous data and the Fisher exact test for nominal data. The 3 subgroups (MALS, immune paralysis and unclassified) were compared with the Kruskal-Wallis test for continuous data and the Fisher-Freeman-Halton test for nominal data. A P-value of <0.05 was considered statistically significant

†statistically significant: healthy controls vs. total sepsis group

‡P-value adjusted by the Bonferroni correction for multiple testing: 0.023 for immune paralysis vs. MALS and 0.021 for unclassified vs. MALS

MALS; macrophage activation-like syndrome, CCI; Charlson comorbidity index, APACHE; acute physiology and chronic health evaluation, NS; not significant, NA; not applicable, SOFA; sequential organ failure assessment, IQR; interquartile range

**Fig. 1:**
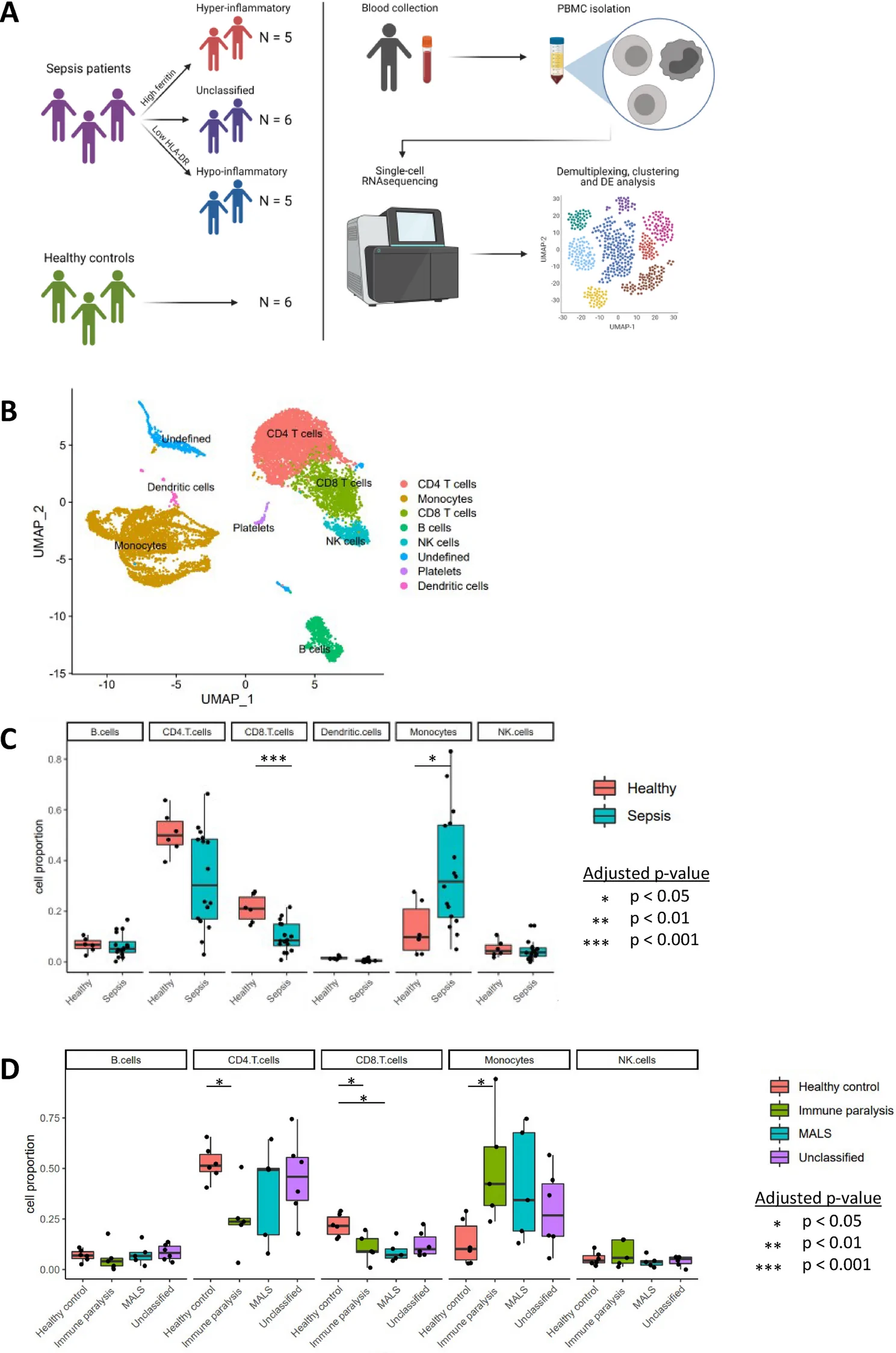
Overview of the study approach and single-cell RNA-sequencing data. (**A**) General overview of the study. Peripheral blood mononuclear cells were isolated from EDTA blood from 16 sepsis patients (5 MALS, 6 unclassified and 5 immune paralysis) and 6 healthy controls, after which single-cell RNA-sequencing was performed using 10X Genomics technology. Created with BioRender.com. (**B**) Uniform manifold approximation and projection (UMAP) plot of 13,059 peripheral blood mononuclear cells from 22 samples (6 healthy controls and 16 sepsis patients). The clusters are annotated based on marker gene expression. (**C-D**) Boxplots of cell proportions for the main cell types, comparing healthy controls to sepsis patients (**C**) or comparing healthy controls to immune paralysis, MALS and unclassified patients (**D**). False discovery rate (FDR) adjusted p-value < 0.05 was considered to be statistically significant

Single-cell RNA-sequencing (scRNA-seq) was performed on PBMCs to characterize the immune signature of sepsis and specific immunological subgroups. One sample was identified as an outlier based on the uniform manifold approximation and projection (UMAP) plot (Supplemental Fig. 1A), and it was therefore excluded. Overall, we have therefore studied biological samples of 16 sepsis patients (5 MALS, 6 unclassified, and 5 immune paralysis) and 6 healthy individuals for further analysis (Fig. 1A).

No major batch effects were observed. After filtering for singlets, number of features per cell and mitochondrial read percentage, more than 13,000 cells across 22 samples remained for analysis. UMAP clustering combined with marker gene expression identified all major cell types expected in the mononuclear blood compartment (Fig. 1B and Supplemental Fig. 1B). Cell subset analysis revealed a shift in circulating immune cell proportions in sepsis, characterized by an increase in monocytes (Dirichlet regression analysis, false discovery rate [FDR] adjusted p-value = 0.04) and a decrease in CD8 T cells (adjusted p-value = 5.5 × 10^− 4^, Fig. 1C). This shift was more pronounced in the immune paralysis group, with reduced CD4 and CD8 T cell proportions (adjusted p-value = 0.04 and adjusted p-value = 0.02, respectively), and an increased monocyte fraction (adjusted p-value = 0.02, Fig. 1D).

### Cell-type specific transcriptomic changes in sepsis

**Fig. 2:**
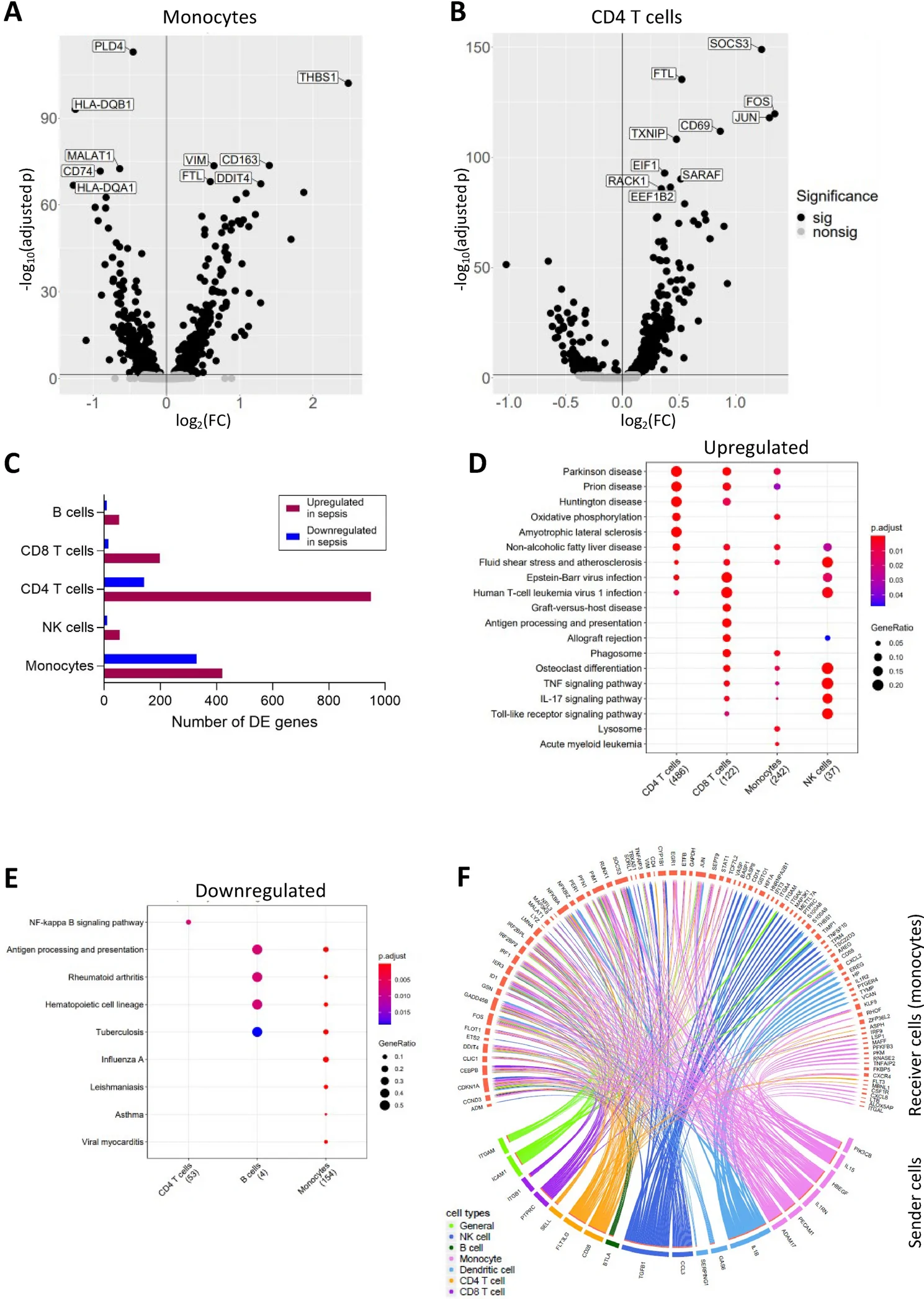
Sepsis-specific immune signatures compared to healthy controls. Volcano plots showing results from differential expression analysis (Wilcoxon rank-sum tests) between sepsis patients and healthy controls in the monocyte (**A**) and CD4 T cell subset (**B**). The -log_10_(Bonferroni adjusted p-value) is visualized on the y-axis (adjusted p-value < 0.05 is considered to be statistically significant) and the log_2_(fold change) is visualized on the x-axis. (**C**) Number of differentially expressed genes per cell type with an adjusted p-value < 0.05. Upregulated genes (higher in sepsis patients compared to healthy controls) are visualized in purple, downregulated genes in blue. (**D**-**E**) Enrichment analyses based on upregulated genes (**D**) or downregulated genes (**E**) using the KEGG database. (**F**) Monocyte-centric ligand-receptor interaction atlas using the NicheNet algorithm with monocyte as the receiver cell type and the other main cell types as sender cells

To define if the dysregulated immune response in sepsis is caused solely by differences in cell proportions or also through changes in transcriptional programs, we compared gene expression signatures between sepsis patients and healthy controls across six main immune cell types (Supplemental Fig. 2). Transcriptomic differences were most noticeable in monocytes (Fig. 2A) and CD4 T cells (Fig. 2B). An overview of the number of differentially expressed genes across cell types is shown in Fig. 2C. In CD4 T cells, 29% of analyzed genes were significantly altered in sepsis (adjusted p-value < 0.05), whereas 12% of genes were differentially expressed in monocytes. Among the most upregulated genes in CD4 T cells of sepsis patients were *SOCS3*, a suppressor of cytokine signaling, and the transcription factors *FOS* and *JUN*, which regulate cell proliferation and differentiation. In monocytes, *THBS1*, a glycoprotein involved in platelet aggregation and angiogenesis, was the most upregulated gene, while HLA-DQA1 and HLA-DQB1, both involved in antigen presentation, were the most downregulated. KEGG pathway enrichment analysis revealed that genes upregulated in sepsis were enriched in pathways involved in oxidative phosphorylation (in CD4 T cells and monocytes) and TNF signaling (in CD8 T cells, monocytes and NK cells; Fig. 2D), and these pathways were also enriched in a sensitivity analysis corrected for age and sex (Supplemental Fig. 3). Downregulated pathways included antigen processing and presentation (in B cells and monocytes; Fig. 2E), which was also observed after adjustment for age and sex (Supplemental Fig. 3). GO terms enrichment analysis revealed enrichment of NADH dehydrogenase activity among upregulated genes in CD4 T cells, while pathways involved in antigen presentation (MHC protein complex binding) were downregulated in B cells and monocytes (Supplemental Fig. 4). Given the high number of differentially expressed genes in monocytes from sepsis patients, we constructed a monocyte-centric ligand-receptor interaction atlas between the main cell types. This analysis revealed extensive interaction between monocytes and all cell types, with most interactions occurring between monocytes themselves (Fig. 2F). We also observed a crosstalk pathway in which the decoy receptor IL-1 receptor type II (*IL1R2*) on monocytes potentially neutralized IL-1β (*IL1B*) from dendritic cells.

### Sepsis patients with MALS or immune paralysis show profound transcriptional differences in monocytes

**Fig. 3:**
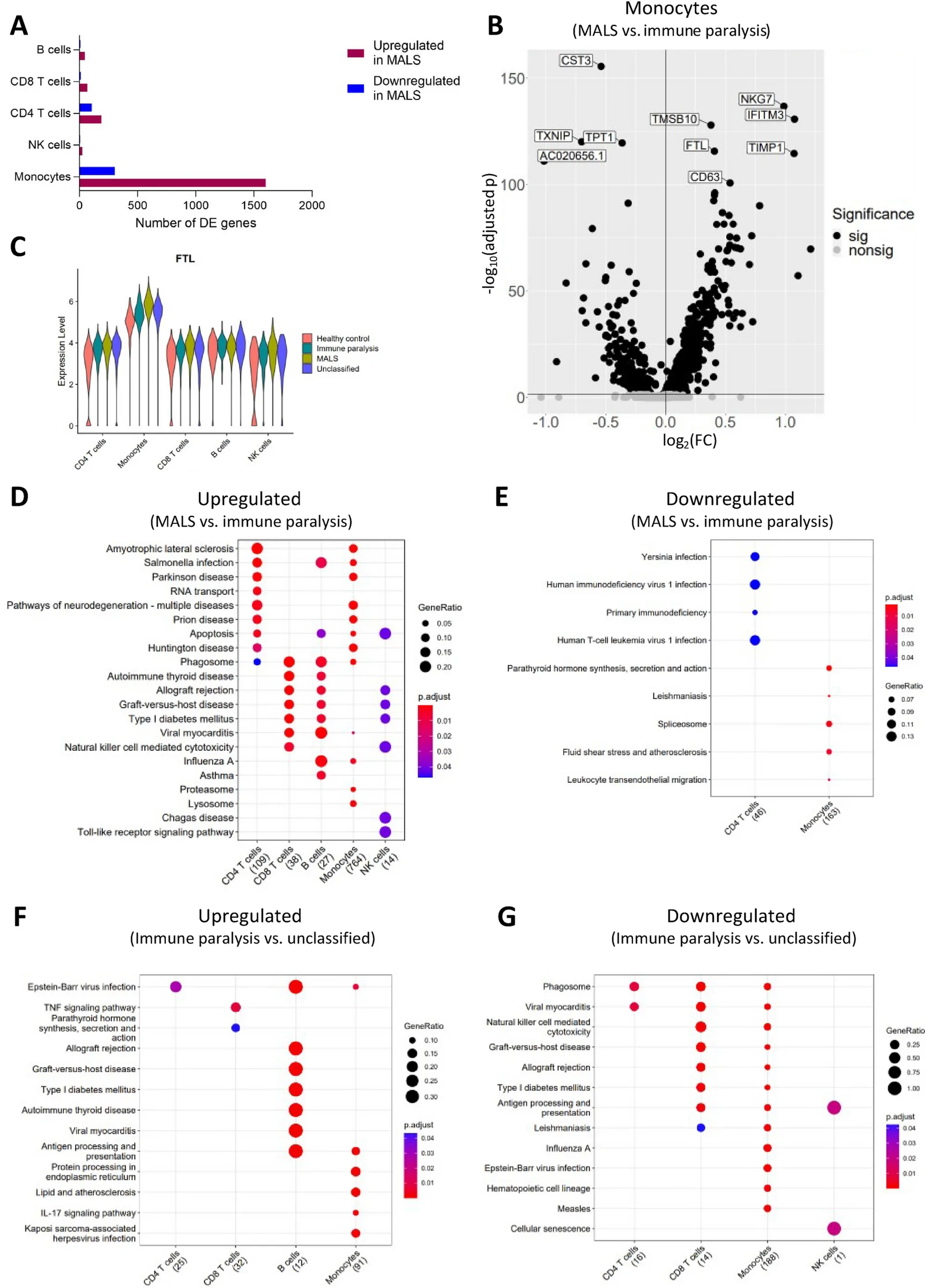
Differences in immune cell-states between MALS and immune paralysis patients. (**A**) Number of differentially expressed genes per cell type. Upregulated genes (higher in MALS patients compared to immune paralysis patients) are visualized in purple, downregulated genes in blue. (**B**) Volcano plot showing the differentially expressed genes (Wilcoxon rank-sum tests) between MALS and immune paralysis patients in monocytes. The -log_10_(Bonferroni adjusted p-value) is visualized on the y-axis (adjusted p-value < 0.05 is considered to be statistically significant) and the log_2_(fold change) is visualized on the x-axis. (**C**) Violin plot of *FTL* expression per cell type separated by group. (**D**-**E**) Enrichment analyses based on upregulated genes (**D**) or downregulated genes (**E**) in MALS compared to immune paralysis patients using the KEGG database. (**F**-**G**) Enrichment analyses based on upregulated genes (**F**) or downregulated genes (**G**) in immune paralysis compared to unclassified patients using the KEGG database

To understand the immunological basis which distinguishes MALS from immune paralysis patients, we compared gene expression in immune cells between MALS and immune paralysis, excluding dendritic cells due to low cell numbers, differentially expressed genes were calculated (Supplemental Fig. 5). The most pronounced differences were observed in monocytes (Fig. 3A). Among the differentially expressed genes in monocytes (Fig. 3B), *FTL*, a subunit of ferritin, was increased in monocytes from MALS compared to immune paralysis patients (Fig. 3C), confirming the high circulating ferritin concentrations that define MALS. Further analysis based on genes higher expressed in MALS revealed an enrichment in apoptosis (in CD4 T cells, B cells, monocytes and NK cells) and phagosome activation (in CD4 T cells, CD8 T cells, B cells and monocytes) pathways (KEGG pathways: Fig. 3D). The GO terms enrichment analysis identified enrichment of NADH hydrogenase activity in monocytes from MALS patients (GO terms: Supplemental Fig. 6). Fewer pathways were enriched among immune paralysis-related genes compared to MALS, and included mainly disease-related pathways e.g. HIV, primary immunodeficiency, T-cell leukemia and parathyroid hormone disease (KEGG pathways: Fig. 3E).

Furthermore, we compared immune paralysis patients to the unclassified group. Enrichment analysis of differentially expressed genes identified IL-17 signaling in monocytes among upregulated genes and NK cell-mediated cytotoxicity in CD8 T cells and monocytes (Fig. 3F and G). Interestingly, antigen processing and presentation and MHC-related pathways were enriched among both upregulated and downregulated genes, indicating active regulation of this pathway in immune paralysis (Fig. 3F and G, and Supplemental Fig. 7).

**Fig. 4:**
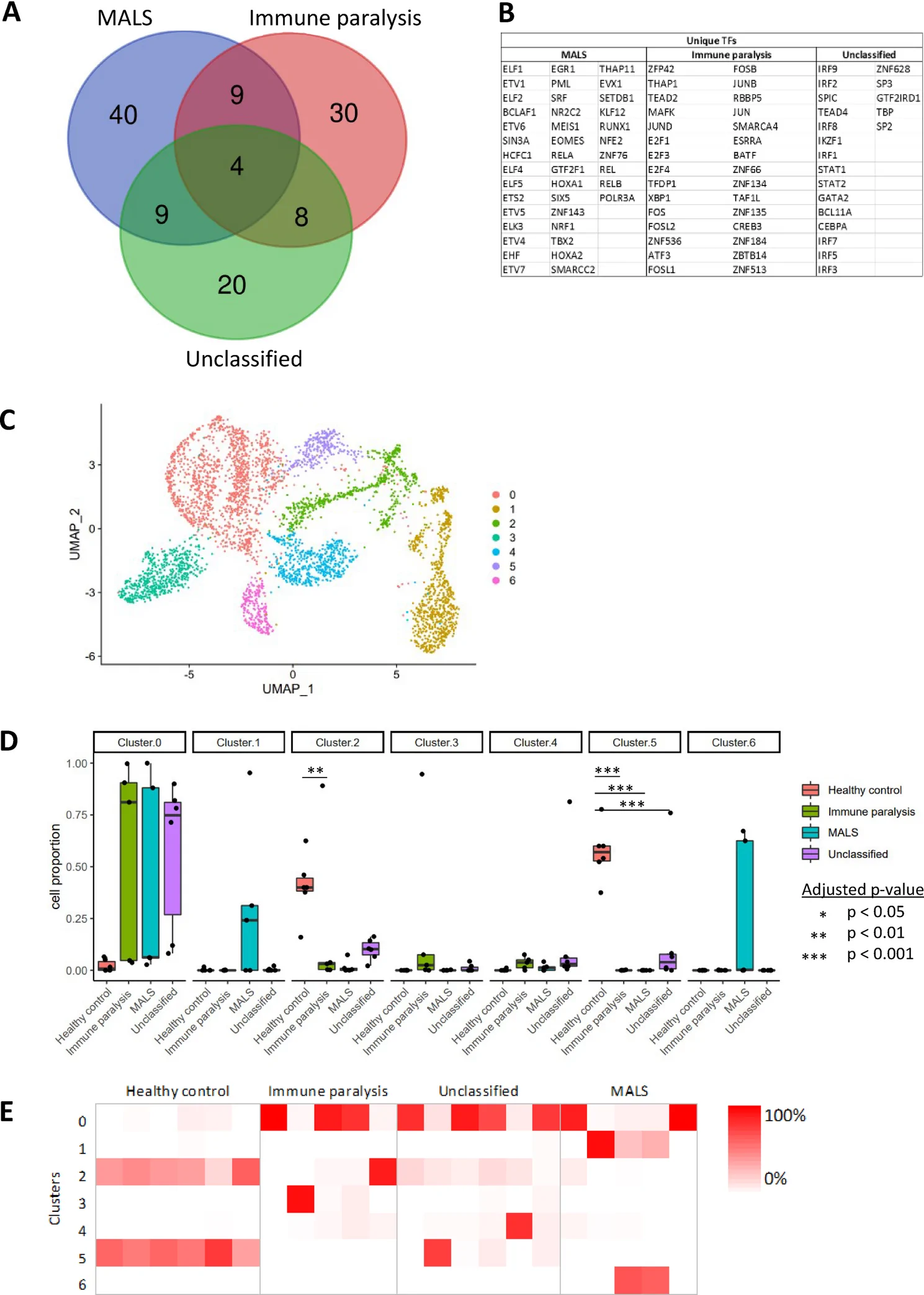
Monocyte signatures and enriched transcription factors in sepsis. (**A-B**) Enriched transcription factors based on differentially expressed genes between conditions in monocytes are visualized in a Venn diagram (**A**). The transcription factors that are uniquely enriched in only one condition are separately listed (**B**). (**C**) Uniform manifold approximation and projection (UMAP) plot of monocytes from 22 samples (6 healthy controls and 16 sepsis patients), identifying seven distinct monocyte clusters. (**D**) Boxplot of cell proportions of the seven monocyte clusters comparing healthy controls to immune paralysis, MALS and unclassified patients. FDR adjusted p-value < 0.05 is considered to be statistically significant. (**E**) Visualization of monocyte cluster proportions among all 22 study participants, divided by endotype

Further, to understand the molecular drivers of the distinct sepsis immune endotypes, we performed a transcription factor (TF) enrichment analysis based on differentially expressed genes comparing monocytes from each endotype to the other two groups. In total, 40 TFs were uniquely enriched in MALS monocytes, 20 in monocytes from the unclassified group, and 30 in immune paralysis monocytes (Fig. 4A). Monocytes from unclassified patients were mainly driven by TFs related to interferon signaling, including *STAT1*, *STAT2*, *IRF2*, *IRF3*, *IRF5*, *IRF7* and *IRF9*. In contrast, immune paralysis monocytes were enriched in TFs from the *JUN* and *FOS* family, regulating monocyte differentiation [37], while MALS monocytes were enriched in *ETS* family TFs, including *ELF1*, *ELF2*, *ETV1* and *ETS2* (Fig. 4B). The enrichment of these distinct transcription factor families shed light on the molecular regulation of monocytes in sepsis.

### Identification of monocyte subclusters related to sepsis and sepsis-specific immune endotypes

Given the heterogeneity in the monocyte population and the large number of differentially expressed genes, we decided to study this subset in more detail. UMAP clustering revealed seven distinct monocyte subsets (Fig. 4C). These subsets were unevenly distributed among sepsis immune endotypes (Fig. 4D and E, and Supplemental Fig. 8). Cluster 0 monocytes were abundant across all three immune endotypes, while clusters 1 and 6 were identified only in MALS. In addition, clusters 2 and 5 were observed more frequently in healthy controls compared with sepsis patients (Dirichlet regression analysis, adjusted p-value < 0.01). Clusters 3, 5 and 6 predominantly consisted of classical monocytes (high *CD14* expression), while clusters 0, 1, 2 and 4 contained a mix of classical and non-classical monocytes (high *CD16* expression, Supplemental Fig. 8). These analyses identify monocyte subsets that are unique to sepsis and to specific immune endotypes present in sepsis.

**Fig. 5:**
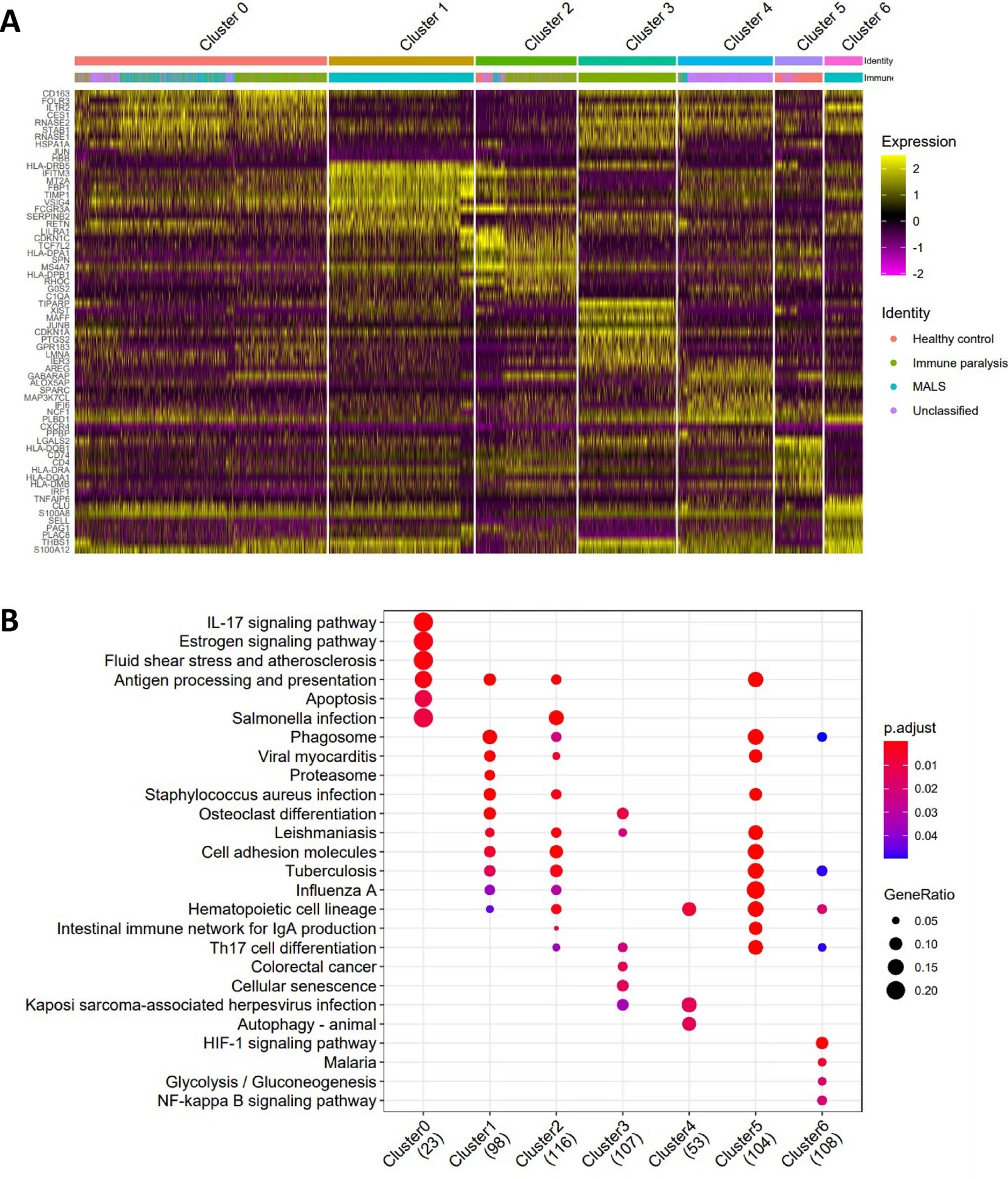
Functional analysis of monocyte subsets. (**A**) Heatmap of the top 10 marker genes of each monocyte cluster. (**B**) Enrichment analysis based on monocyte cluster marker genes using the KEGG database

Since these monocyte subsets vary across sepsis immune endotypes, we aimed to understand differences in their transcriptional programs. Analysis of cluster-specific marker genes showed that several HLA genes, including *HLA-DRB5*, *HLA-DPB1*, *HLA-DQB1*, and *HLA-DMB*, distinguished the monocyte populations (Fig. 5A and Supplemental Fig. 9). Pathway analysis of marker genes revealed that cluster 0 monocytes are characterized by genes related to IL-17 signaling and apoptosis (Fig. 5B). In addition, cluster 6 monocytes are characterized by genes associated with HIF-1 signaling and glycolysis. Additional enriched pathways across clusters include antigen processing and presentation (cluster 0, 1, 2, and 5), phagosome (cluster 1, 2, 5, and 6), and hematopoietic cell lineage (cluster 1, 2, 4, 5, and 6).

### Identification of monocyte cluster signatures in an in-vitro endotoxin tolerance model

**Fig. 6:**
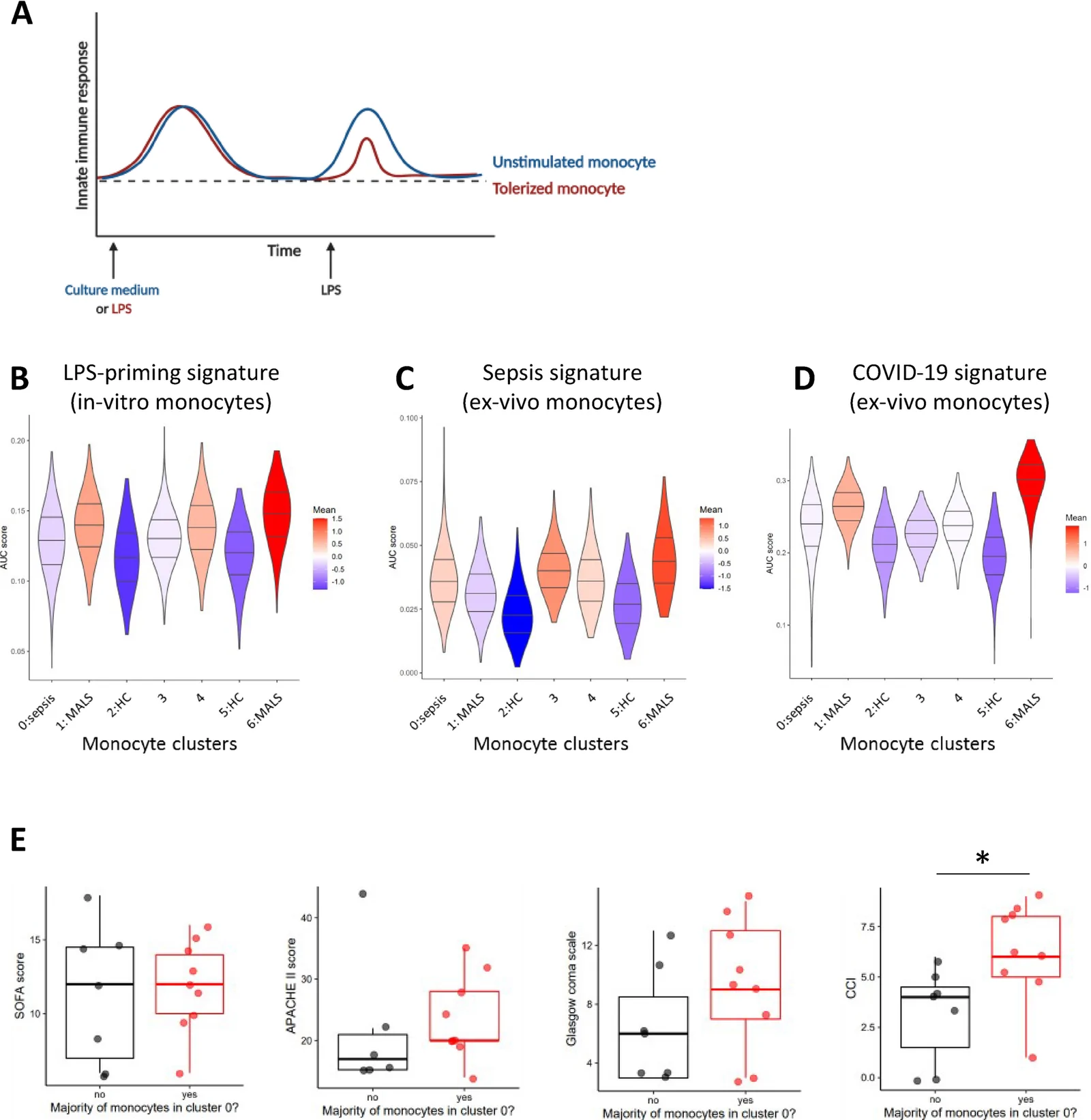
Validation of monocyte subsets and linkage to disease. A graphical overview of LPS-induced tolerance in monocytes in vitro (**A**). A gene set enrichment analysis to test for the enrichment of gene signatures of an in-vitro LPS tolerance model (**B**), bacterial sepsis (**C**) and severe COVID-19 (**D**) in the marker genes of the monocyte subsets identified in this sepsis cohort. The area under the curve (AUC) values are visualized in violin plots. (**E**) Boxplots comparing four disease severity markers (the Sequential Organ Failure Assessment [SOFA] score, the Acute Physiology and Chronic Health Evaluation [APACHE] II score, and the Charlson Comorbidity Index [CCI]) between sepsis patients having the majority of their monocytes as part of cluster 0 versus sepsis patients who do not have the majority of their monocytes in cluster 0. * p-value < 0.05

To explore the role of the novel monocyte subpopulations, we linked them to relevant cell signatures from experimental models. For this, we performed an additional study using an experimental model of immune paralysis induced by endotoxin tolerance in human primary monocytes [38] (Fig. 6A). Monocytes were exposed to LPS for 24 h to induce tolerization, followed by a 5-day resting period and subsequent restimulation with LPS for 4 h. Transcriptome profiles were measured using single cell SORT-seq (35). Differentially expressed genes between tolerant (LPS pre-exposed) and control (unexposed/naive) monocytes after LPS restimulation were determined, and enrichment of this signature was calculated across monocyte subsets (Fig. 6B). Interestingly, the in-vitro LPS-induced immune tolerance signature was strongly present in cluster 6 monocytes, suggesting MALS is also associated with poor immune responsiveness. This is consistent with the concept that sepsis involves in some patients concurrent excessive inflammation and immune suppression [39].

### Validation of monocyte cluster signatures in urosepsis and COVID-19 patients

Furthermore, we assessed the signature of a previously reported sepsis-specific monocyte cluster described in urinary tract infection–associated sepsis [30]. This signature was enriched most prominently in cluster 6, which was associated with MALS (Fig. 6C). Cluster 3 also showed enrichment of this signature and was mainly observed in the immune paralysis group. However, because this signal was driven largely by one immune paralysis patient, validation in larger cohorts will be needed to determine whether cluster 3 represents a reproducible feature of immune paralysis. This demonstrates once more the immune heterogeneity of sepsis, and that the anatomical/etiologic stratification of sepsis patients is insufficient to identify immune endotypes and guide immunotherapy. Interestingly, both the in-vitro LPS immune tolerance signature and the bacterial sepsis signature were absent in cluster 2 and 5, mainly present in healthy controls.

Next, we analyzed a monocyte signature specific for severe COVID-19, based on genes upregulated in monocytes from patients with severe compared with mild disease [31]. Interestingly, the signature of severe COVID-19 overlapped with transcriptional profiles of cluster 1 and 6 monocytes (Fig. 6D), both MALS-specific monocyte clusters, indicating shared monocyte responses between severe COVID-19 and MALS. Indeed, the hyperinflammatory character of COVID-19 is now well established [40–42], and immunotherapy with anti-IL-1 or anti-IL-6 treatment has proven successful [43–46].

Finally, we aimed to better understand the clinical relevance of monocyte subcluster 0, which is present in all immune endotypes of sepsis patients. As shown in Fig. 4E, sepsis patients can be divided into two groups based on the proportion of cluster 0 monocytes: those with a high (> 70%) or low (< 15%) fraction. We compared these groups using three clinical indices: the Sequential Organ Failure Assessment (SOFA) score, the Acute Physiology and Chronic Health Evaluation (APACHE) II score, and the Charlson Comorbidity Index (CCI; Fig. 6E). For APACHE II, there is a clear trend visible, where the cluster 0 monocyte population was linked to a higher score. For CCI, this was statistically significant (Wilcoxon rank-sum test, p-value < 0.05, adjusted p-value = 0.07). These data suggest that the sepsis-specific monocyte cluster 0 probably mirrors the response to severe infections in individuals with many comorbidities.

## Discussion

Sepsis immunotherapy remains challenging due to the clinical and immunological heterogeneity of patients. MALS and immune paralysis are well-characterized sepsis immune endotypes, defined by distinct and accessible biomarkers and associated with patient outcomes [17]. Although the ImmunoSep trial highlighted the potential of precision immunotherapy targeting these immune endotypes [18], the biological mechanisms underlying them remain poorly understood. To our knowledge, this is the first single-cell transcriptomic study to specifically characterize circulating immune cell programs in biomarker-selected MALS and immune paralysis patients. By profiling immune cells at single-cell resolution, we identified distinct monocyte transcriptional programs, regulatory pathways, and monocyte subclusters associated with these immune endotypes. These findings provide mechanistic insight into MALS and immune paralysis and may support further patient stratification and personalized immunotherapy.

Defects in innate and adaptive immune cells are proposed to drive (mal)adaptive immune responses in sepsis [47]. In line with previous findings, our cell proportion analysis revealed decreased lymphocyte subsets, particularly CD8 and CD4 T cells, in sepsis patients. The decrease in CD4 T cells was most pronounced in patients classified as immune paralysis, indicating that not solely innate immunity is compromised (low monocytic HLA-DR expression), but also adaptive immunity is impaired in these patients, reflecting two important aspects in the pathogenesis of immune paralysis [47].

Comparing transcriptional profiles of sepsis patients with healthy controls revealed pronounced differences in CD4 T cell and monocyte subsets. Cytokine signaling pathways (TNF and IL-17 signaling) were increased in CD8 T cells, monocytes and NK cells, while oxidative phosphorylation was increased in the CD4 T cells and monocytes, suggesting increased metabolic activity. Enrichment of cytokine signaling pathways aligns with previous studies, which describe roles for TNF-α and IL-17 in sepsis pathophysiology [48, 49]. Cell-cell interaction analysis further indicated that monocytes mainly respond to ligands from other monocyte subpopulations, suggesting a positive feedback activation loop that could support host defense, but may overshoot in MALS if exaggerated.

The pathogenesis of MAS/MALS is still not completely understood. In genetically-mediated MAS, defective lymphocyte cytolytic activity leads to persistent immune activation [50–52]. In contrast, our data show that transcriptional differences between MALS and other immune endotypes are especially driven by monocyte alterations, rather than lymphoid cells. This finding that myeloid cell hyperactivation is likely responsible for excessive inflammatory mediator release in MALS is consistent with observations that exaggerated activity of IL-1/IL-18 characterizes hyperinflammation in sepsis.

In contrast, immune paralysis is characterized by reduced monocytic HLA-DR expression and defective responsiveness of myeloid and lymphoid cells to microbial stimulation [53]. Compared with immune paralysis, immune cells from MALS patients showed enrichment of genes linked to apoptosis in NK cells, CD4 T cells and B cells, and phagosome activation in CD4 T cells, CD8 T cells, B cells and monocytes. Lymphocyte apoptosis contributes to immune dysregulation in sepsis [54], indicating that apoptosis inhibitors could potentially support immune function. However, our observed upregulation of apoptosis pathways appears limited to MALS patients, suggesting the need to stratify patients to identify MALS when considering therapies targeting apoptosis.

Monocytes exhibited the highest number of differentially expressed genes between MALS and immune paralysis. Based on previous data [5], our study used ferritin concentrations (> 4,420 ng/mL) to identify MALS. We validated this concept at a gene-transcriptional level by high expression of *FTL*, a subunit of ferritin, in monocytes, likely reflecting iron accumulation in myeloid cells in early sepsis [55]. Pathway analysis further showed upregulated genes related to intracellular infections (e.g. influenza A and salmonella), hinting towards the interferon pathway, and increased NADH dehydrogenase activity. Together, these changes suggest that transcriptional reprogramming of monocytes may play a central role in MALS pathogenesis.

In the unclassified group, we show enrichment of STAT/IRF regulators, related to interferon signaling. These patients have a better outcome [17], suggesting that proper interferon pathway activation is necessary for adequate host defense. This is consistent with IFN-β restoring monocyte function in endotoxemia models [56], and IFN-γ improving organ dysfunction in sepsis [18].

We identified specific monocyte subsets associated with sepsis immune endotypes. Two clusters (clusters 1 and 6) were uniquely enriched in MALS and absent in other immune endotypes and healthy controls. Cluster 6 showed enrichment of metabolic dysregulation targeting glucose metabolism and HIF-1α pathways, consistent with reports linking increased *HIF1A* expression to sepsis non-survivors [57]. These findings support the concept that metabolic dysregulation of immune cells contributes to sepsis pathophysiology [58–60] and restoration of metabolism in MALS could represent a promising therapeutic target. Notably, comparison with an independent urosepsis cohort demonstrated overlap with the MALS-associated monocyte cluster 6 and cluster 3 in our dataset, which may reflect an immune paralysis-associated monocyte state. Sepsis due to urogenital infections was not included in our cohort, highlighting that transcriptional programs can overlap regardless of the origin of infection.

Finally, we identified a sepsis-specific monocyte cluster (cluster 0) associated with higher CCI scores and a trend toward higher APACHE II scores, independent of the underlying endotype. Since both CCI and APACHE II are linked to mortality in sepsis [61, 62], cluster 0 may reflect a monocyte state associated with greater comorbidity burden and overall clinical vulnerability in sepsis. This cluster was identified in an independent cohort of severely ill sepsis patients. IL-17 signaling was enriched in cluster 0, potentially contributing to inflammation and tissue damage in sepsis [48]. Consistent with this observation, elevated IL-17 concentrations are associated with increased mortality risk in sepsis [63], suggesting that IL-17 may represent a therapeutic target in selected patients.

Our study has also a few limitations. First, one limitation is the relatively small sample size, increasing sensitivity to individual patient effects. Because this study was imbedded in an ongoing multi-center study, the collected samples represented the maximum number available at the enrolling center with the required technical expertise. Nevertheless, the sample size is in line with other single-cell RNA-sequencing studies in sepsis that have identified transcriptomic patterns in cohorts of comparable size [20, 57, 64]. MALS is a rare immune endotype, occurring only in 5% of sepsis patients and 20% of patients with septic shock [5, 17]. Despite its higher prevalence (~ 40%), large screening cohorts were required to identify patients with immune paralysis. The unclassified group should not be interpreted as a distinct validated immune endotype, but rather as a heterogeneous group of sepsis patients who did not meet the predefined biomarker criteria for either MALS or immune paralysis. This group was included to represent an intermediate immune-status group and to provide a comparison population between the two predefined immune endotypes. Second, the cross-sectional design limits insight into the dynamic immune response during sepsis. Longitudinal multi-omics studies will be needed to characterize temporal changes in endotypes, underscored by the observed instability of biomarker-based endotypes in sepsis, with patients rapidly changing endotypes within 48 h of ICU admission [14]. Third, the in-vitro model of LPS tolerance cannot fully mimic the in-vivo situation of patients with immune paralysis. Finally, neutrophils were not captured in the single-cell RNA-sequencing dataset. The study design did not allow for this, because samples were selected post-hoc from cryopreserved PBMCs after biomarker-based classification into MALS or immune paralysis, and the technical difficulty for preserving neutrophils. Although our findings have been validated in independent cohorts of urosepsis and COVID-19 patients, as well as in a model of experimental LPS-induced tolerance, more validation studies are needed.

## Conclusions

We report heterogeneity of monocyte populations in critically ill sepsis patients with different immune endotypes ranging from MALS to immune paralysis. We identified a sepsis-specific monocyte subset linked to greater comorbidity burden and increased clinical vulnerability in sepsis, as well as MALS-specific monocyte clusters that could represent the basis for personalized therapeutic targeting. Understanding and identifying monocyte signatures underlying immune endotypes offers a promising approach to stratify patients for precision medicine in sepsis.

## Supplementary Information

Below is the link to the electronic supplementary material.

Supplementary Material 1

## Data Availability

Code generated to process the data are freely available on Github (https://github.com/CiiM-Bioinformatics-group/Sepsis). All data will be made available on the European Genome-Phenome Archive (EGA; ega-archive.org). The accession code is being generated and will be added once it becomes available.

## Abbreviations

APACHE: Acute Physiology and Chronic Health Evaluation
AUC: Area Under the Curve
BSA: Bovine Serum Albumin
COVID: Coronavirus Disease
DGE: Digital Gene Expression
DMSO: Dimethyl Sulfoxide
DNA: Deoxyribonucleic Acid
EDTA: Ethylenediaminetetraacetic acid
FBS: Fetal Bovine Serum
FDR: False Discovery Rate
HIF: Hypoxia-Inducible Factor
HIV: Human Immunodeficiency Virus
HLA: Human Leukocyte Antigen
ICU: Intensive Care Unit
IFN: Interferon
IL: Interleukin
IQR: Interquartile Range
KEGG: Kyoto Encyclopedia of Genes and Genomes
LPS: Lipopolysaccharide
MACS: Magnetic-Activated Cell Sorting
MHC: Major Histocompatibility Complex
MALS: Macrophage Activation-Like Syndrome
MAS: Macrophage Activation Syndrome
NADH: Nicotinamide Adenine Dinucleotide
PBMC: Peripheral Blood Mononuclear Cell
PBS: Phosphate Buffered Saline
RNA: Ribonucleic Acid
RPMI: Roswell Park Memorial Institute (medium)
SOFA: Sequential Organ Failure Assessment
SNN: Shared Nearest Neighbor
TNF: Tumor Necrosis Factor
UMAP: Uniform Manifold Approximation and Projection
